# Liquid Chromatography-Tandem Mass Spectrometry of Desoxo-Narchinol a and Its Pharmacokinetics and Oral Bioavailability in Rats and Mice

**DOI:** 10.3390/molecules24112037

**Published:** 2019-05-28

**Authors:** Subindra Kazi Thapa, Mahesh Upadhyay, Tae Hwan Kim, Soyoung Shin, Sung-Joo Park, Beom Soo Shin

**Affiliations:** 1 Department of Pharmacy, College of Pharmacy, Wonkwang University, Iksan, Jeonbuk 54538, Korea; thapasubindra@gmail.com (S.K.T.); maheshupadhyay@gmail.com (M.U.); shins@wku.ac.kr (S.S.); 2 College of Pharmacy, Daegu Catholic University, Gyeongsan, Gyeongbuk 38430, Korea; thkim@cu.ac.kr; 3 Department of Herbology, School of Korean Medicine, Wonkwang University, Iksan, Jeonbuk 54538, Korea; parksj08@wku.ac.kr; 4 Hanbang Cardio-Renal Syndrome Research Center, Wonkwang University, Iksan, Jeonbuk 54538, Korea; 5 School of Pharmacy, Sungkyunkwan University, Suwon, Gyeonggi 16419, Korea

**Keywords:** desoxo-narchinol A, Nardostachys jatamansi, LC-MS/MS, pharmacokinetics, bioavailability

## Abstract

Desoxo-narchinol A is one of the major active constituents from *Nardostachys jatamansi*, which has been reported to possess various pharmacological activities, including anti-inflammatory, antioxidant, and anticonvulsant activity. A simple and sensitive liquid chromatography-tandem mass spectrometry (LC-MS/MS) method was developed and validated for the quantification of desoxo-narchinol A in two different biological matrices, i.e., rat plasma and mouse plasma, using sildenafil as an internal standard (IS). The method involved simple protein precipitation with acetonitrile and the analyte was separated by gradient elution using 100% acetonitrile and 0.1% formic acid in water as a mobile phase. The MS detection was performed with a turbo electrospray in positive ion mode. The lower limit of quantification was 10 ng/mL in both rat and mouse plasma. Intra- and inter-day accuracies were in the ranges of 97.23–104.54% in the rat plasma and 95.90–110.11% in the mouse plasma. The precisions were within 8.65% and 6.46% in the rat and mouse plasma, respectively. The method was applied to examine the pharmacokinetics of desoxo-narchinol A, and the oral bioavailability of desoxo-narchinol A was 18.1% in rats and 28.4% in mice. The present results may be useful for further preclinical and clinical studies of desoxo-narchinol A.

## 1. Introduction

*Nardostachys jatamansi* is a pharmacologically versatile herb found in alpine Himalayas [1]. Traditionally, the roots of *Nardostachys jatamansi* have been used as an aromatic, bitter tonic, antispasmodic, stimulant, antiseptic, diuretic, and emmenagogue [2,3]. The pharmacological activities of *Nardostachys jatamansi* have been well demonstrated in experimental animals. For example, studies have reported that *Nardostachys jatamansi* possesses hepato-protective [1], gamma-aminobutyric acid (GABA) enhancing [4], anti-parkinsonian [5], and anticonvulsant [6] activities in rats. Various activities of *Nardostachys jatamansi*, including antidepressant [7], improvement of learning and memory [8], antidiabetic [9], and anti-inflammatory [10], have also been demonstrated in mice. Recently, in-vitro-derived plants of *Nardostachys jatamansi* have also shown an anti-cholinesterases, anti-hyperglycemic, anti-inflammatory, anti-hypertensive, and anti-tyrosinase potential [11].

The pharmacological activities of *Nardostachys jatamansi* might be attributed to the various compounds present in *Nardostachys jatamansi*, including sesquiterpenes, lignans, and neolignans, terpinic coumarins, phenols, flavonoids, and alkaloids [3,12]. Desoxo-narchinol A is a nardosinone-type sesquiterpenoid found in the rhizomes and roots of *Nardostachys jatamansi*. Desoxo-narchinol A has shown protective effects against lipopolysaccharide (LPS)-induced inflammation through p38 deactivation [13]. Inhibitory activity of desoxo-narchinol A against LPS-induced nitric-oxide (NO) production has been reported as well [14]. Recently, it has also been reported that desoxo-narchinol A inhibited the excessive production of pro-inflammatory mediators and cytokines in LPS-stimulated BV2 and primary microglial cells [15,16]. Therefore, desoxo-narchinol A may be useful as a potential therapeutic agent for the treatment of inflammation or prevention of neurodegenerative diseases. 

Despite extensive research on its pharmacological activities, there has been limited information regarding the pharmacokinetics of *Nardostachys jatamansi* or its active ingredient, desoxo-narchinol A. To better understand the biological actions and the therapeutic effects for further development as a therapeutic agent, comprehensive pharmacokinetic studies based on robust analytical methods are essential. To date, a liquid chromatography-tandem mass spectrometry (LC-MS/MS) of nardosinone, another active ingredient of *Nardostachys jatamansi*, has been developed and applied to characterize its pharmacokinetics following intravenous injection in rats [17]. For desoxo-narchinol A, there is only one recent pharmacokinetic study after oral administrations of desoxo-narchinol A and extracts of *Nardostachys jatamansi* in rats by applying an LC-MS/MS method [18]. However, the LC-MS/MS utilized solid-phase extraction, which requires long sample preparation time and cost. Moreover, the pharmacokinetics were only determined following oral administration in rats [18]. Its oral bioavailability and comprehensive pharmacokinetic characteristics are still unknown. The potential differences in pharmacokinetics in different animal species that have been utilized for the pharmacological studies need to be elucidated as well.

Therefore, the aim of the present study was to develop and validate a simple, rapid, and sensitive LC-MS/MS analysis for the quantification of desoxo-narchinol A in the biological fluids. The method was successfully applied to determine the oral bioavailability and pharmacokinetics of desoxo-narchinol A in two animal species, i.e., rats and mice, following intravenous and oral administration. To our knowledge, this is the first study investigating the comprehensive pharmacokinetics of desoxo-narchinol A in rats as well as in mice.

## 2. Results and Discussion

### 2.1. Optimization of Sample Preparation

Sample pretreatment procedure was needed to remove protein and potential interferences before LC-MS/MS analysis. Various sample extraction techniques have been applied to extract analytes from biological samples, including protein precipitation, liquid–liquid extraction, and solid–phase extraction. Since the protein precipitation provides a simple and rapid method to extract analytes from the biological matrices [19,20], we applied protein precipitation to prepare samples in the present study. Different organic solvents, namely methanol and acetonitrile, were investigated as protein precipitation solvents to achieve good resolution and high recovery of analytes from spiked biological matrices. Finally, a direct protein precipitation method using acetonitrile was found to be optimal and was selected for biological sample preparation. The main advantages of the present method include the simplicity and low cost of the single-step protein precipitation over other extraction methods. Although solid-phase extraction has been effectively used to extract desoxo-narchinol A and nardosinonediol from plasma [18], it involves greater complexity, lengthy sample preparation time, and higher cost.

### 2.2. Chromatography

**Figure 1:**
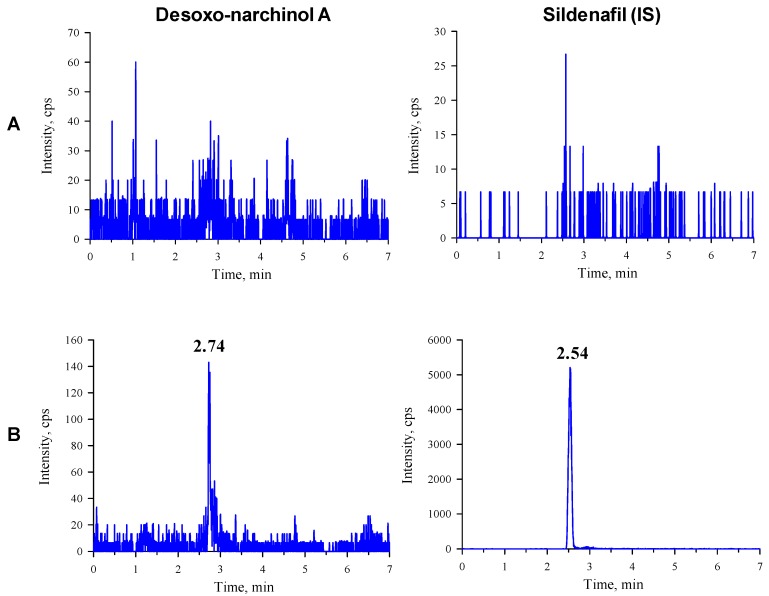
Representative chromatograms of desoxo-narchinol A (left) and the internal standard (right) in the (**A**) blank rat plasma and (**B**) blank rat plasma spiked with desoxo-narchinol A (LLOQ) and IS.

**Figure 2:**
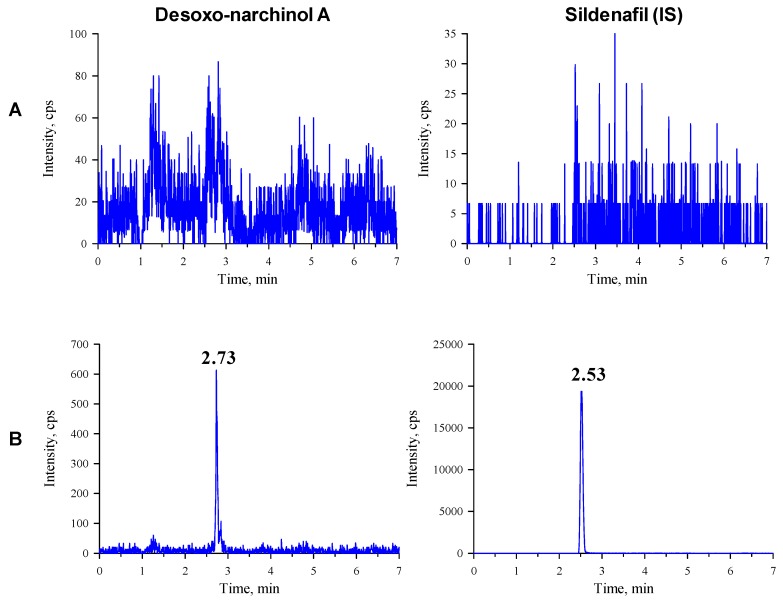
Representative chromatograms of desoxo-narchinol A (left) and the internal standard (right) in the (**A**) blank mouse plasma and (**B**) blank mouse plasma spiked with desoxo-narchinol A (LLOQ) and IS.

The observed multiple reaction monitoring (MRM) transitions and assay parameters for desoxo-narchinol A and sildenafil (internal standard, IS) are summarized in Table 1. The representative chromatograms in the rat and mouse plasma are shown in Figure 1 and Figure 2, respectively. Desoxo-narchinol A and IS were eluted for 2.74 min and 2.54 min, respectively, in the rat plasma. Similarly, the retention time of desoxo-narchinol A was 2.73 min and that of IS was 2.53 min in the mouse plasma. The complete chromatographic run took 7 min. 

Specificity is the ability to determine accurately and specifically the analyte in the presence of other components that may be expected to be present in the matrix. Specificity was assessed by comparing the chromatograms of the blank rat and mouse plasma with the blank matrix spiked with the analyte and IS. Examination of blank, zero sample, and other calibrators showed no interfering peaks at the retention times corresponding to desoxo-narchinol A or IS. 

### 2.3. Linearity and Sensitivity

Linearity refers to the ability to obtain test results that are proportional to analyte concentration within a given range. The calibration curves for desoxo-narchinol A in both rat and mouse plasma were linear in the concentration range of 10–1000 ng/mL with correlation coefficients (r^2^) > 0.999. The calibration curves had a reliable reproducibility over the standard concentrations of the analyte across the calibration range. The lower limit of quantification (LLOQ) was defined as the lowest standard concentration on the calibration curve with an accuracy of 80–120% and a precision less than 20% [21]. The LLOQ of desoxo-narchinol A was 10 ng/mL for both rat and mouse plasma, which provided sufficient sensitivity to characterize its pharmacokinetics following intravenous and oral administration. 

### 2.4. Accuracy, Precision, and Recovery

The accuracy indicates the closeness between the measurement and the true or theoretical value, and precision is the closeness among a series of measurements. Assessment of accuracy and precision of the analytical method is essential to determine whether the method is ready for validation [21]. The present method was validated in two different biological matrices, i.e., rat and mouse plasma, by using matrix matched quality control (QC) samples to demonstrate its accuracy and precision. The intra- and inter-day accuracy and precision were assessed by analyzing five replicates of LLOQ (10 ng/mL), and QC samples at three different concentrations (25, 125, and 800 ng/mL) in the blank rat and mouse plasma each day for five days. According to the FDA guidance [21], the mean accuracy should not deviate by ±15 %, except for LLOQ, where it can be ±20% of the nominal concentration. Similarly, the deviation at each concentration level from the nominal concentration was expected to be within ± 15%, except LLOQ, for which it should not be more than ± 20% [21]. 

The determined intra- and inter-day accuracy and precision are summarized in Table 2. The intra- and inter-day accuracies were 97.23–104.54% and the precisions were within 8.65% in the rat plasma. In the mouse plasma, the intra- and inter-day accuracies were 95.90–110.11% and the precisions were within 6.46%. The intra- and inter-day accuracy and precision of the present assay satisfied the criteria of the FDA guidance on bioanalytical methods validation [21] and indicated that the established method was accurate and reliable.

The total recovery of desoxo-narchinol A that was determined by comparing the peak responses of the drug free plasma or Milli-Q water spiked with desoxo-narchinol A is summarized in Table 3. Although recovery does not need to be 100%, it has been recommended that the extent of recovery of an analyte and IS should be consistent and reproducible [21]. The average recovery for desoxo-narchinol A was 95.15–99.10% in the rat plasma and 95.53–100.07% in the mouse plasma, indicating that the extraction of desoxo-narchinol A via a single-step protein precipitation was efficient and reproducible. 

### 2.5. Stability

Analyte stability in a given matrix should be determined during sample collection, processing, and storage of the analysis to ensure that the analytical method generates reliable data. The stability was evaluated by comparing the peak responses of the QC samples of desoxo-narchinol A at two different concentrations (25 and 800 ng/mL) in the rat or mouse plasma that were stored in four different conditions against those of freshly prepared QC samples. Results of autosampler stability, freeze-thaw stability, short-term stability, and long-term stability are shown in Table 4. As shown in Table 4, the stability QC samples displayed average 96.62–104.98% recoveries in the rat plasma and 97.43–106.11% in the mouse plasma. Any significant deviations were not observed compared to the freshly prepared samples, indicating that there was no significant degradation of desoxo-narchinol A under the tested conditions. 

### 2.6. Pharmacokinetics of Desoxo-Narchinol A in Rats

The developed method was successfully applied to pharmacokinetic studies of desoxo-narchinol A following intravenous and oral administration in two animal species. 

**Figure 3:**
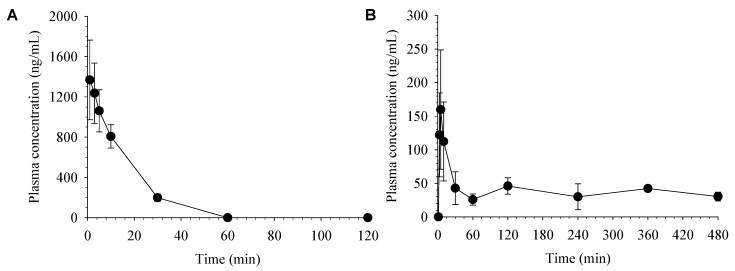
Average plasma concentration vs. time profiles of desoxo-narchinol A after (A) intravenous administration at a dose of 5 mg/kg (*n* = 4) and (B) oral administration at a dose of 50 mg/kg (*n* = 4) in rats. Data represent the mean ± SD.

The average plasma concentration-time profiles of desoxo-narchinol A after intravenous and oral administration in rats are shown in Figure 3. The corresponding pharmacokinetic parameters of desoxo-narchinol A calculated via non-compartmental analysis are shown in Table 5. 

In rats, following intravenous injection, plasma concentrations of desoxo-narchinol A were declined with the elimination half-life (t_1/2_) of 10.2 ± 0.7 min and not detected after 60 min. Following oral administration, desoxo-narchinol A was rapidly absorbed, reached the maximum plasma concentration (C_max_) at 5 min. Then, the plasma concentration of desoxo-narchinol A showed a multi-exponential decline with an extended terminal phase, resulting in the prolonged terminal t_1/2_ compared to that after intravenous injection. Multiple peaks were observed in most of the individual plasma concentration-time profiles. Due to the presence of secondary peaks in the plasma concentration-time profiles, terminal phase could not be easily defined and t_1/2_, area under the plasma concentration-time curve (AUC_inf_), and apparent clearance (CL/F) were estimated from the limited number of animals. The rapid absorption and prolonged terminal t_1/2_ after oral absorption is consistent with the recent literature report, which reported the T_max_ and t_1/2_ of desoxo-narchinol A of 7.5 ± 2.7 min and 248.8 ± 135.2 min, respectively, following oral administration in rats [18]. The extended terminal phases of desoxo-narchinol A was also observed following oral administration of extracts from *Nardostachys jatamansi* [18]. Finally, the oral bioavailability of desoxo-narchinol A was estimated at 18.1% in rats.

Extensive enterohepatic recirculation typically leads to multiple peaks or shoulders in the plasma concentration-time profiles and a prolonged terminal half-life [22,23]. Alternatively, complex absorption processes, including the presence of different absorption sites in the gastrointestinal tract, may also be associated with the double peak phenomenon [24]. Although multiple peaks and prolonged terminal half-lives were observed only after oral administration, further studies are required to elucidate its mechanisms including enterohepatic recirculation, which may have a pronounced impact on the systemic exposure, and thus on the pharmacological effects of desoxo-narchinol-A. 

### 2.7. Pharmacokinetics of Desoxo-Narchinol A in Mice

**Figure 4:**
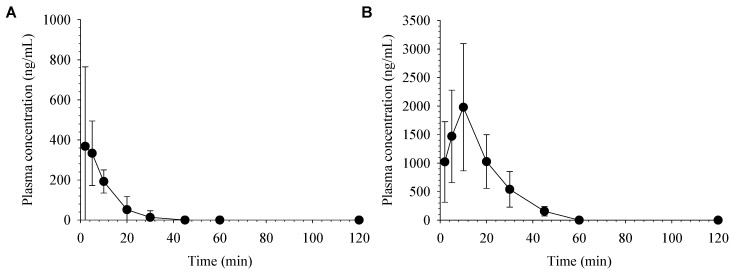
Average plasma concentration vs. time profiles of desoxo-narchinol A after (A) intravenous administration at a dose of 2 mg/kg (*n* = 6) and (B) oral administration at a dose of 50 mg/kg (*n* = 5) in mice. Data represent the mean ± SD.

Figure 4 depicts the average plasma concentration-time profiles of desoxo-narchinol A after intravenous and oral administration in mice. The non-compartmental pharmacokinetic parameters of desoxo-narchinol A in mice are summarized in Table 5. Similar to the pharmacokinetics in rats, desoxo-narchinol A was rapidly disappeared from the plasma with t_1/2_ of 7.4 ± 5.0 min and not detected after 45 min following intravenous injection in mice. Following oral administration, desoxo-narchinol A was rapidly absorbed to reach C_max_ within 10 min and decreased with the elimination t_1/2_ of 9.8 ± 2.3 min. The extended terminal t_1/2_ and multiple peaks were not observed in mice either after intravenous or oral administration. The species differences in the pharmacokinetic disposition may be worthy of further studies. The oral bioavailability of desoxo-narchinol A was 30.9% in mice, which was higher than that in rats. To best of our knowledge, this is the first report of oral bioavailability of desoxo-narchinol A in animals.

## 3. Materials and Methods 

### 3.1. Materials 

Desoxo-narchinol A was supplied by the School of Oriental Medicine, Wonkwang University (Iksan, South Korea) and stored at −20 °C. Sildenafil (IS), dimethyl sulfoxide (DMSO), and formic acid were purchased from Sigma-Aldrich, Inc. (St. Louis, MO, USA). Acetonitrile and methanol were the products of Burdick and Jackson (Muskegon, MI, USA). All other reagents were high-performance liquid chromatography (HPLC) grades. Water used during the entire study was purified using a Milli-Q water purification system. 

### 3.2. Animal Study 

The animal studies were approved by the Institutional Animal Care and Use Committee (IACUC) at Wonkwang University (WKU17-02) and conducted following the Guidelines for the Care and Use of Animals. Male Sprague-Dawley (SD) rats (7 weeks old) and ICR (CD-1) mice (7 weeks old) were purchased from Hanil sirhamdongmul center (Wanju, South Korea). Animals were kept in plastic cages with free access to standard diet and water. The animals were maintained at a temperature of 22–24 °C with a 12 h light-dark cycle and relative humidity of 50 ± 10%.

#### 3.2.1. Rat Study

Rats were anesthetized with intraperitoneal injection of 20 mg/kg Zoletil 50^®^ (tiletamine HCl 125 mg/5 mL + zolazepam HCl 125 mg/5 mL) and cannulated with a polyethylene tubing (0.58 mm i.d., 0.96 mm o.d., Natsume, Tokyo, Japan) in the right jugular vein. After 24 h of recovery, the rats were examined for their physical condition and the experiment was carried out only if the animal was found to be stable. After overnight fasting, desoxo-narchinol A dissolved in 10% DMSO was administered to rats by intravenous (IV, 5 mg/kg, *n* = 4) injection or oral (PO, 50 mg/kg, *n* = 4) administration. Blood samples (200 μL) were collected from the jugular vein before and at 1, 3, 5, 10, 30 min, 1, and 2 h after IV injection, and at 1, 3, 5, 10, 30 min, 1, 2, 4, 6, and 8 h after oral administration. Plasma samples were harvested by centrifugation of the blood samples at 4,000× *g* for 10 min and stored at −20 °C until analysis.

#### 3.2.2. Mice Study

Mice were administered with desoxo-narchinol A by IV injection into the caudal vein (2 mg/kg, *n* = 6) or oral administration (50 mg/kg, *n* = 5). Blood samples (approximately 40 μL) were collected from the retro-orbital sinus before and at 2, 5, 10, 20, 30, 45 min, 1, and 2 h after drug administration. By centrifugation of the blood samples at 4,000 × g for 10 min, plasma samples were stored at −20 °C until analysis.

#### 3.2.3. Noncompartmental Analysis

The pharmacokinetic parameters of desoxo-narchinol A were determined by noncompartmental analysis using the Phoenix^®^ WinNonlin^®^ (Certara, L.P., Princeton, NJ, USA). The apparent terminal half-life (t_1/2_) was calculated as 0.693/*λ_z_*, where *λ_z_* is the terminal slope. The area under the plasma concentration-time curve from time zero to the last observation time point (AUC_all_) was calculated using the trapezoidal method and AUC to infinity (AUC_inf_) was obtained by adding C_last_/*λ_z_* to AUC_all_. The systemic clearance (CL) and apparent clearance (CL/F) were estimated by Dose/AUC_inf_. Volume of distribution at steady state (V_ss_) was calculated as CL·MRT, where MRT is the mean residence time. Maximum plasma concentration (C_max_) and time to reach C_max_ (T_max_) were obtained directly from the observed data. The oral bioavailability (F) was calculated as F = (AUC_inf, oral_·Dose_iv_)/(AUC_inf, iv_·Dose_oral_).

### 3.3. Calibration Standards and Quality Control Samples

The standard stock solutions of desoxo-narchinol A and IS were prepared in methanol at the concentration of 1.0 mg/mL. The working standard solutions of desoxo-narchinol A were prepared by serial dilution of the stock solution with acetonitrile, yielding concentrations of 10, 50, 100, 250, 500, and 1000 ng/mL. The IS working solution 200 ng/mL was prepared by dilution of the stock solution with acetonitrile. 

To prepare calibration standard samples, blank rat or mouse plasma were diluted 10-fold with water. Then, 50 μL of the diluted plasma were spiked with 50 μL of the IS working solution and 50 μL of the desoxo-narchinol A standard working solution. Acetonitrile 100 μL was added to the mixture as a protein precipitation solvent. The mixture was mixed on a vortex mixer for 1 min followed by centrifugation at 16,000× *g* for 20 min at 4 °C. The supernatant 10 μL was injected onto the LC-MS/MS. 

The matrix-matched QC samples were prepared by spiking the desoxo-narchinol A working solutions to the freshly harvested blank rat or mouse plasma to provide LLOQ (10 ng/mL), low concentration QC (25 ng/mL), medium concentration QC (125 ng/mL), and high concentration QC (800 ng/mL). The QC samples were stored at −20 °C until analysis. 

To prepare plasma samples, the obtained plasma samples were diluted 10-fold with water and 50 μL of the diluted plasma samples were mixed with 150 μL of acetonitrile and 50 μL of the IS solution (200 ng/mL) on a vortex mixer. After the mixture was centrifuged at 16,000× *g* for 20 min at 4 °C, the supernatant was collected and 10 μL was injected onto the LC-MS/MS.

### 3.4. LC-MS/MS Conditions

LC-MS/MS system consisted of API 2000 triple quadrupole mass spectrometer (AB Sciex, Concord, ON, Canada) coupled with a Nanospace, Shiseido HPLC system (Shiseido, Yokohama, Japan). Plasma samples were separated on a Kinetex C_18_ column (100 × 2.10 mm i.d., 5 μm). The composition of mobile phase was a mixture of 0.1% of formic acid in water (mobile phase A) and acetonitrile (mobile phase B) with gradient elution program set as: 10% B (0 → 0.5 min), 10% → 90% B (0.5 → 1 min), 90% B (1 → 4.5 min), 90% → 10% B (4.5 → 5 min), and 10% B (5 → 7 min). The flow rate was 0.2 mL/min and the temperature of the autosampler and column were set to be 4 °C and 40 °C, respectively. 

The electrospray ionization (ESI) source was operated in positive mode with the curtain and turbo-gas (all nitrogen) set at 30 and 6 psi, respectively. The turbo-gas temperature and the ion spray needle voltage were set at 400 °C and 4500 V, respectively. The mass spectrometer was operated in the MRM mode with a dwell time of 150 ms per MRM channel. The selected precursor/product ion pairs were *m*/*z* 192.94 → 99.00 for desoxo-narchinol A and *m*/*z* 474.76 → 58.1 for IS. The collision energy was set at 19 and 67 eV for desoxo-narchinol A and IS, respectively. Table 1 summarizes the observed MRM transitions and mass spectrometry settings for desoxo-narchinol A and IS. Data acquisition was performed with Analyst 1.4 software (AB Sciex).

### 3.5. Assay Validation

#### 3.5.1. Specificity, linearity, and Sensitivity

Specificity was evaluated by analyzing the blank matrix and the blank matrix spiked with the analyte and IS. The linearity was assessed over the concentration ranges of 10–1000 ng/mL in both rat and mouse plasma. The calibration curves were constructed by the weighted regression method (1/x) of peak area ratios of desoxo-narchinol A to IS compared to the actual concentration. The determination of r^2^ > 0.999 was considered desirable for the calibration curve. The lowest standard concentration on the calibration curve was accepted as the LLOQ. The analyte peak of LLOQ sample should be identifiable, discrete, and reproducible with accuracy within ±20% and precision ≤20%. The deviation of standards of other than LLOQ from the nominal concentration should be within ±15%.

#### 3.5.2. Accuracy and Precision

The accuracy, precision, and recovery were assessed by using the matrix-matched LLOQ and QC samples. Intra- and inter-day accuracies and precisions were expressed as a percent of deviation from the respective nominal values. The intra-day accuracy and precision of QC samples were determined within one day. The inter-day accuracy and precision of the QC samples were determined on five different days. 

#### 3.5.3. Recovery

The total recovery of the analyte at LLOQ and three QC samples were determined by analyzing samples prepared by spiking desoxo-narchinol A in drug-free plasma and Milli-Q water separately, and that spiked in Milli-Q water served as un-extracted QC samples. The recovery of desoxo-narchinol A and IS were determined by using five replicates and were processed as usual and analyzed along with five replicates of un-extracted QC samples. The extraction recovery of analyte was determined by measuring the peak area ratios of the analyte after extraction of plasma samples to those of un-extracted QC samples.

#### 3.5.4. Stability

The stability of desoxo-narchinol A was examined under four different conditions using five replicates of low (25 ng/mL) and high (800 ng/mL) matrix-matched QC samples. To assess the stability of desoxo-narchinol A in the rat and mouse plasma at room and storage temperature, low and high QC samples were left at 20 °C for 4 h and at −20 °C for 2 weeks, respectively, and desoxo-narchinol A concentrations were determined. The autosampler storage stability was determined by storing the QC samples in the autosampler at 4 °C for 24 h before being analyzed. The freeze-thaw stability was assessed by determining the remaining concentrations after low and high QC samples were subjected to three freeze-thaw cycles. The results were expressed as the percentage of the mean calculated over theoretical concentrations

## 4. Conclusions

In this study, a simple and rapid LC-MS/MS method for the analysis of desoxo-narchinol A was developed and validated in two biological matrices—rat plasma, and mouse plasma. The assay involves small sample volumes and single-step protein precipitation resulting in simple and sensitive analysis of desoxo-narchinol A with the LLOQ of 10 ng/mL in both rat and mouse plasma. The developed method was fully validated to demonstrate its reproducibility, as well as specificity, sensitivity, accuracy, precision, recovery, and stability. By applying the LC-MS/MS method, the pharmacokinetics and oral bioavailability of desoxo-narchinol A were determined after intravenous and oral administration in two animal species. The LC-MS/MS assay and the pharmacokinetic information of desoxo-narchinol A in rats and mice may provide useful information for further preclinical and clinical studies of desoxo-narchinol A.

## Figures and Tables

**Table 1:** Observed MRM transitions and mass spectrometry settings.

Compounds	MRM Transition (*m*/*z*)	Retention Time (min)		DP (V)	FP (V)	CE (eV)	CXP (V)
		Rat Plasma	Mouse Plasma				
Desoxo-narchinol A	192.94 → 99.00	2.74	2.73	56	310	19	2
IS	474.76 → 58.10	2.54	2.53	96	370	67	0

Note: DP, declustering potential; FP, focusing potential; CE, collision energy; CXP, collision cell exit potential.

**Table 2:** Intra- and inter-day accuracy and precision of desoxo-narchinol A assay in rat and mouse plasma.

Matrix	Concentration (ng/mL)	Intra-Day (*n* = 5)		Inter-Day (*n* = 5)	
		Accuracy (%)	Precision (%)	Accuracy (%)	Precision (%)
Rat plasma	10	97.23	8.65	104.54	8.30
	25	99.80	3.90	100.81	1.97
	125	102.24	5.26	99.54	1.66
	800	102.64	1.10	98.28	3.23
Mouse plasma	10	98.41	1.59	110.11	6.46
	25	97.81	0.67	104.20	3.64
	125	97.31	0.78	100.85	2.13
	800	95.90	0.46	99.93	2.93

**Table 3:** Total recovery (%) for desoxo-narchinol A and IS in rat and mouse plasma (*n* = 5).

	Concentration (ng/mL)	Rat Plasma (%)	Mouse Plasma (%)
Desoxo-narchinol A	10	98.77 ± 3.18	99.37 ± 0.65
	25	99.10 ± 2.00	100.07 ± 0.88
	125	95.15 ± 1.63	95.53 ± 1.33
	800	96.10 ± 1.56	96.22 ± 1.29
IS	200	103.90 ± 2.32	100.12 ± 3.48

**Table 4:** Stability of desoxo-narchinol A in blank rat and mouse plasma (*n* = 5).

Matrix	Conc (ng/mL)	Percentage over Theoretical Concentration (%)			
		Autosampler Stability (24 h, 4 °C)	Freeze-Thaw Stability (3 cycles, −20 °C)	Short-Term Stability (4 h, 20 °C)	Long-Term Stability (2 wk, −20 °C)
Rat plasma	25	102.34 ± 1.75	96.62 ± 0.89	98.23 ± 1.63	104.98 ± 1.05
	800	99.06 ± 1.76	98.45 ± 1.15	99.68 ± 1.49	102.86 ± 1.66
Mouse plasma	25	104.24 ± 0.50	106.11 ± 0.70	105.23 ± 0.49	98.61 ± 0.84
	800	99.59 ± 0.43	103.46 ± 0.80	100.00 ± 0.56	97.43 ± 1.07

**Table 5:** Non-compartmental pharmacokinetic parameters of desoxo-narchinol A following intravenous injection (IV) or oral administration (PO) in rats and mice.

Pharmacokinetic Parameters	Rat		Mouse	
	IV (5 mg/kg, *n* = 4)	PO (50 mg/kg, *n* = 4)	IV (2 mg/kg, *n* = 6)	PO (50 mg/kg, *n* = 5)
t_1/2_ (min)	10.2 ± 0.7	516.9 ± 99.4 ^a^	7.4 ± 5.0	9.8 ± 2.3
T_max_ (min)	-	5.0 ± 0.0	-	10.0 ± 0.0
C_0_ or C_max_ (ng/mL)	1442.5 ± 463.6	159.8 ± 88.8	624.9 ± 434.8	1978.5 ± 1114.4
AUC_all_ (ng·h/mL)	399.8 ± 73.0	317.9 ± 46.6	80 ± 25.8	688.9 ± 279.5
AUC_inf_ (ng·h/mL)	400.8 ± 73.1	725.1 ± 213.3 ^a^	102.2 ± 27.2	726.6 ± 303.4
CL or CL/F (mL/min/kg)	213.8 ± 39.3	950.1 ± 574.0 ^a^	343.9 ± 83.1	1302.5 ± 505.3
V_ss_ (L/kg)	2.9 ± 0.6	-	3.6 ± 2.7	-
Bioavailability (F)	-	18.1%	-	28.4%

Note: ^a^, *n* = 3; t_1/2_, terminal half-life; T_max_, time to reach C_max_; C_0_ or C_max_, maximum plasma concentration after i.v. or oral administration, respectively; AUC_all_, area under the plasma concentration-time curve from time zero to the last observation time point; AUC_inf_, AUC to infinity; CL or CL/F, systemic clearance or apparent clearance; V_ss_, volume of distribution at steady state.
